# Influence of Diabetes-Induced Glycation and Oxidative Stress on the Human Rotator Cuff

**DOI:** 10.3390/antiox11040743

**Published:** 2022-04-08

**Authors:** Tomoya Yoshikawa, Yutaka Mifune, Atsuyuki Inui, Hanako Nishimoto, Kohei Yamaura, Shintaro Mukohara, Issei Shinohara, Ryosuke Kuroda

**Affiliations:** Department of Orthopaedic Surgery, Kobe University Graduate School of Medicine, 7-5-2 Kusunoki-cho, Chuo-ku, Kobe 650-0017, Japan; tomo.yoshi.0926@gmail.com (T.Y.); ainui@med.kobe-u.ac.jp (A.I.); hanakoni@med.kobe-u.ac.jp (H.N.); kyamaura@sprivail.org (K.Y.); no.8shintaro@gmail.com (S.M.); 203m878m@stu.kobe-u.ac.jp (I.S.); kurodar@med.kobe-u.ac.jp (R.K.)

**Keywords:** rotator cuff, glycation reaction, advanced glycation end-products, oxidative stress, reactive oxygen species, diabetes, hyperglycemia

## Abstract

Most shoulder rotator cuff tears (RCTs) are caused by non-traumatic age-related rotator cuff degeneration, of which hyperglycemia is a risk factor due to its glycation reaction and oxidative stress. We aimed to identify the influence of diabetes-induced glycation and oxidative stress in patients with non-traumatic shoulder RCTs. Twenty patients, aged over 50 years, with non-traumatic shoulder RCTs participated in this study. Patients with a history of diabetes mellitus or preoperative HbA1c ≥ 6.5% were assigned to the diabetic group (*n* = 10), and the rest to the non-diabetic group (*n* = 10). Cell proliferation; expression of genes related to oxidative stress, glycation reaction, inflammation, and collagen; intracellular reactive oxygen species (ROS) levels; and apoptosis rates were analyzed. The diabetic group had significantly lower cell proliferation than the non-diabetic group. In the diabetic group, the mRNA expression levels of *NOX1*, *NOX4*, *IL6*, *RAGE*, type III collagen, *MMP2*, *TIMP1*, and *TIMP2* were significantly higher; type I collagen expression was significantly lower; and the rate of ROS-positive cells and apoptotic cells, as well as the expression of advanced glycation end-products (AGEs) and the receptor for AGEs (RAGE), was significantly higher. In conclusion, hyperglycemia caused by diabetes mellitus increased AGE and RAGE expression, and led to increased NOX expression, ROS production, and apoptosis in the human rotator cuff. This provides scope to find a preventive treatment for non-traumatic RCTs by inhibiting glycation and oxidative stress.

## 1. Introduction

The causes of shoulder rotator cuff tears can be broadly classified as traumatic and non-traumatic [1]. Most common is non-traumatic age-related rotator cuff degeneration, and hyperglycemia is the one of the risk factors, due to its glycation reaction and associated oxidative stress [2]. Diabetes mellitus (DM) is a condition of high blood glucose levels [3]. High-blood-glucose-induced excessive oxidative stress can cause tissue damages and organ malfunction [4]. Intracellular reactive oxygen species (ROS) levels, the main cause of oxidative stress, increase under high-glucose conditions [5]. Increased ROS cause DNA, RNA, and protein damage and changes in antioxidant enzyme levels, eventually damaging cells and tissues [4,5]. Reports in the past have shown that the main cause of hyperglycemia-induced ROS is nicotinamide adenine dinucleotide phosphate oxidase (NOX), as its activation enhances the production of ROS [6,7,8]. Moreover, protein glycation is involved with mechanisms of oxidative stress under hyperglycemic conditions [9]. Glycation reaction, or irreversible glucose binding on protein amino groups, generates advanced glycation end-products (AGEs) [9]. AGEs combine with the receptor for AGEs (RAGE), a specific receptor, to activate NOX, thereby leading to the development of oxidative stress and the inflammatory state [10]. AGE accumulation increases in aging or diabetic patients, and is associated with tissue damage, leading to the development of diabetic complications [11]. Additionally, AGEs are involved in age-related rotator cuff degeneration [12].

The risk of developing musculoskeletal diseases is higher in patients with DM than in patients without DM [13]. DM is associated with an increase in the prevalence of general shoulder pathology [14]. Shoulder disorders, including shoulder pain, rotator cuff tendinopathy, and rotator cuff tendinitis, have been reported to be 5-fold more prevalent in diabetes patients [15]. Consistent with this, diabetes can also be related to a greater risk of tendinitis [16], with potential functional changes [17,18] and structural abnormalities, including collagen disorganization [19], thickening [16,17,20], and calcification [21], all of which may contribute to an increased risk of tendon rupture [22]. However, it has not been determined whether the severity and duration of diabetes affect the severity of rotator cuff tears. Additionally, the effects of glycation reaction and oxidative stress induced by hyperglycemia on rotator-cuff-derived cells and rotator cuff tissue remain unclear. To address this gap in understanding, we evaluated the influence of diabetes-induced glycation and oxidative stress on the rotator cuffs of patients with non-traumatic shoulder rotator cuff tears.

## 2. Materials and Methods

### 2.1. Ethics Statement

All experiments were conducted under the approval and guidance of the Ethics Committee of our institute, and informed consent was obtained from all patients.

### 2.2. Experimental Protocol

**Figure 1:**
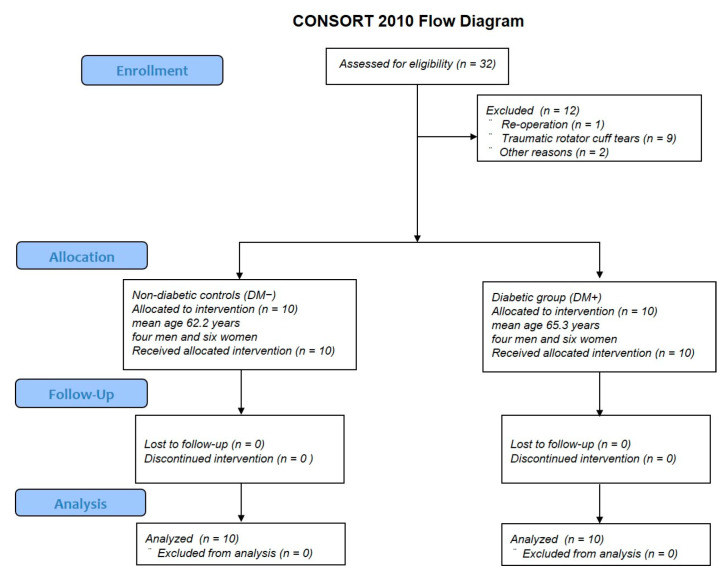
CONSORT flow diagram.

Patients with reoperation, trauma, rheumatoid arthritis, or those aged below 50 years were excluded from this study (Figure 1). The participants of this study included 20 patients with non-traumatic shoulder rotator cuff tears aged over 50 years old, with an average age of 63.8 ± 6.0 years (range, 51–72 years). Patients with a history of diabetes mellitus or preoperative HbA1c ≥ 6.5% were assigned to the diabetic group (DM+), and the rest were assigned to the non-diabetic controls (DM−). The sample size was determined by power analysis based on data from a previous study using G*Power 3.1 [12]. A prior sample size calculation showed the difference in detecting ROS in the high- and low-AGE groups (*n* = 8 per group) using a t-test (effect size = 0.8, α = 0.05, power = 0.95). Patient backgrounds were compared in terms of age, sex, HbA1c levels, prevalence of hypertension, BMI, rotator cuff tear size, and fatty degeneration of the rotator cuff between the two groups. Rotator cuff tear size was defined as small tears (<1 cm), medium tears (1–3 cm), large tears (3–5 cm), and massive tears (>5 cm), according to the Cofield classification [23]. Fatty degeneration was defined based on the Goutallier classification, with stage 0 indicating no fatty infiltration; stage 1, some fatty streaks in the supraspinatus; stage 2, less fat than muscle; stage 3, equal amounts of fat and muscle; and stage 4, more fat than muscle [24]. The correlation between HbA1c levels and duration of diabetes and the size of rotator cuff tears or the degree of fatty degeneration was subanalyzed within the diabetic group.

### 2.3. Preparation of Human Rotator-Cuff-Derived Cells and Tissue

Human rotator cuff tissues were harvested from the torn edges of the supraspinatus tendons during arthroscopic rotator cuff repair. Rotator-cuff-derived cells were isolated by aseptically sectioning the tissue into small pieces of approximately 1.5–2.0 mm^3^. Subsequently, digestion was performed in Dulbecco’s modified Eagle’s medium (DMEM; Sigma, St. Louis, MO, USA) supplemented with 30 mg/mL collagenase II (Gibco, Big Cabin, OK, USA) at 37 °C, 95% humidity, and 5% CO_2_ for 4 h. Following digestion, the cells were pelleted, rinsed twice with phosphate-buffered saline (PBS), and cultured in DMEM (HyClone, Logan, UT, USA) supplemented with 2% fetal bovine serum (Sigma) and 1% penicillin-streptomycin (Sigma). The explants were incubated at 37 °C in a humidified atmosphere of 5% CO_2_. The cells from the rotator cuff were then subcultured after trypsin digestion, and the medium was changed every 3–5 days. In this study, all experiments were performed with one or two passages of cells, and the same passage of cells was used for each experiment. All results were compared between the diabetic and non-diabetic groups.

### 2.4. Cell Proliferation Assay

Cell proliferation was evaluated based on the water-soluble tetrazolium salt (WST) assay using the Cell Counting Kit-8 (Dojindo, Kumamoto, Japan). A total of 5000 cells in 100 µL of DMEM were seeded in all wells in the 96-well plates and incubated at 37 °C under a 5% CO_2_ atmosphere for 48 h. A total of 10 µL of WST was added to each well, followed by incubation of the plates for further 4 h at 37°C under a 5% CO_2_ atmosphere for the WST assay. Formazan conversion from WST was measured spectrophotometrically at 450 nm. The cells in the non-diabetic group were used as controls.

### 2.5. Quantitative Reverse Transcription Polymerase Chain Reaction (qRT-PCR) Analysis

Rotator-cuff-derived cells were seeded onto 12-well culture plates at a density of 1 × 10^5^ cells per well and incubated in DMEM. After 48 h of incubation, total RNA was extracted from each group of cells using the RNeasy Mini Kit (Qiagen, Valencia, CA, USA) in accordance with the manufacturer’s instructions. Total RNA was reverse-transcribed into single-strand complementary DNA using a high-capacity complementary DNA reverse transcription kit (Applied Biosystems, Foster City, CA, USA), according to the manufacturer’s instructions. Thereafter, using the Applied Biosystems 7900HT fast real-time PCR system and SYBR Green reagent (Applied Biosystems), real-time PCR was performed in triplicate to analyze mRNA expression levels of *NOX1*, *NOX4*, interleukin 6 (*IL6*), *RAGE*, type I collagen, type III collagen, matrix metalloproteinase 2 (*MMP2*), tissue inhibitor of matrix metalloproteinase 1 (*TIMP1*), and *TIMP2*. Table 1 shows the used primer sequences; we do not have gene accession number for the custom primers procured from Thermo Fisher Scientific Inc (Waltham, MA, USA). Gene expression was normalized based on the mRNA levels of the housekeeping gene (*GAPDH*), and the results are represented by the 2^−ΔΔCt^ method, comparing those of cells in the non-diabetic group as controls.

### 2.6. Detection of ROS

Rotator-cuff-derived cells were seeded 1 × 10^5^ per well in 12-well plates in 1 mL DMEM and incubated at 5% CO_2_ and 37 °C for 48 h. To examine the intracellular ROS accumulation in rotator-cuff-derived cells, the Total ROS/Superoxide Detection Kit (Enzo Life Sciences, Farmingdale, NY, USA) was used with the oxidation-sensitive fluorescent probe 2′7′-dichlorofluorescin diacetate (DCFH-DA), following protocols provided by the manufacturer. In brief, a final concentration of 10 µM of DCFH-DA was used to treat rotator-cuff-derived cells, which were incubated in the dark at 37 °C for 60 min, washed thrice with PBS, trypsinized, and then resuspended. Using a fluorescence microscope (BZ-X710, KEYENCE, Osaka, Japan), ROS-positive and 2-(4-amidinophenyl)-1H-indole-6-carboxamidine (DAPI)-positive cells in four rectangular areas (0.75 mm × 1.0 mm) on each slide were counted and their mean values were analyzed. The ROS-positive cell rate (number of ROS-positive nuclei/DAPI-positive nuclei) was compared between the two groups.

### 2.7. Cell Apoptosis Analysis

Rotator-cuff-derived cells were seeded in 12-well plates with 1 mL DMEM and incubated for 48 h at 37 °C with 5% CO_2_. Using the APO-DIRECT^TM^ Kit (Phoenix Flow Systems, San Diego, CA, USA), nuclear fragmentation was detected in fixed cells (4% paraformaldehyde/PBS) with terminal deoxynucleotidyl transferase dUTP nick end labeling (TUNEL) staining following protocol of the manufacturer. For counterstaining the nuclei, DAPI solution was used. Using a fluorescence microscope BZ-X710 (KEYENCE), apoptosis-positive and DAPI-positive cells in four rectangular areas (0.75 mm × 1.0 mm) on each slide were counted and their mean values were analyzed. The apoptosis-positive cell rate (number of apoptosis-positive nuclei/DAPI-positive nuclei) was compared between the two groups.

### 2.8. Rotator Cuff Histology and Immunohistochemistry

Each group of 10 formalin-fixed rotator cuff tissues was dehydrated after 24 h and embedded in paraffin wax. Rotator cuff tissues were sequentially sectioned at a thickness of 5 μm. After hematoxylin and eosin staining (H&E), the fiber structure, fiber arrangement, nuclear morphology, and regional variations in cellularity were assessed by a semiquantitative grading scale [25]. A score of 0 to 3 was used to evaluate each variable, with 0 = normal, 1 = slightly abnormal, 2 = abnormal, and 3 = significantly abnormal. Rotator cuff tissues with H&E staining were graded according to five randomly selected optical fields for each tissue section. An analysis of each field was conducted by two blind investigators. In addition, to assess the expression of AGEs and RAGE, anti-AGE (6D12; mouse monoclonal to AGEs, FUJIFILM Wako Pure Chemical, Osaka, Japan), and anti-RAGE antibodies (ab3611; rabbit polyclonal to RAGE, Abcam, Cambridge, UK) were used for immunohistochemical staining. Briefly, the sections were deparaffinized with xylene, dehydrated using graded ethanol, and then incubated with 10 μg/mL of anti-AGEs or anti-RAGE antibodies (both 1:200) overnight at 4 °C. Following incubation with the primary antibody, sections were incubated with a secondary antibody and counterstained with hematoxylin. The signals of AGEs and RAGE were detectable as red color formed by incubation with the peroxidase substrate 3,3′-diaminobenzidine (Nichirei Bioscience, Tokyo, Japan). Digital images were taken using a microscope (BZ-X710, KEYENCE), and the stained areas were quantified using ImageJ software (version 1.53e; National Institutes of Health, Bethesda, MD, USA). The percentage of each positively stained region was evaluated in five optic fields selected at random per histological section, and the mean value was calculated and compared between each group.

### 2.9. Statistical Analysis

All data are presented as the mean ± standard deviation (SD). SPSS (version 27.0; SPSS Inc., Chicago, IL, USA) was used for all statistical analyses. Significant differences between groups were determined using independent *t*-tests and chi-square tests. Statistical significance was set at *p* < 0.05.

## 3. Results

### 3.1. Patient Background

Patient background characteristics are summarized in Table 2. All patients in the diabetic group are those with type 2 diabetes. We observed no significant difference in age, sex, prevalence of hypertension, BMI, rotator cuff tear size, and fatty degeneration between the groups. However, HbA1c levels were significantly higher in the diabetic group (DM−, 5.7 ± 0.26; DM+, 6.9 ± 0.50; *p* < 0.001; Table 2). Moreover, there was no significant correlation between HbA1c levels and the duration of diabetes and the size of rotator cuff tears or the degree of fatty degeneration within the diabetic group.

### 3.2. Cell Proliferation Assay

**Figure 2:**
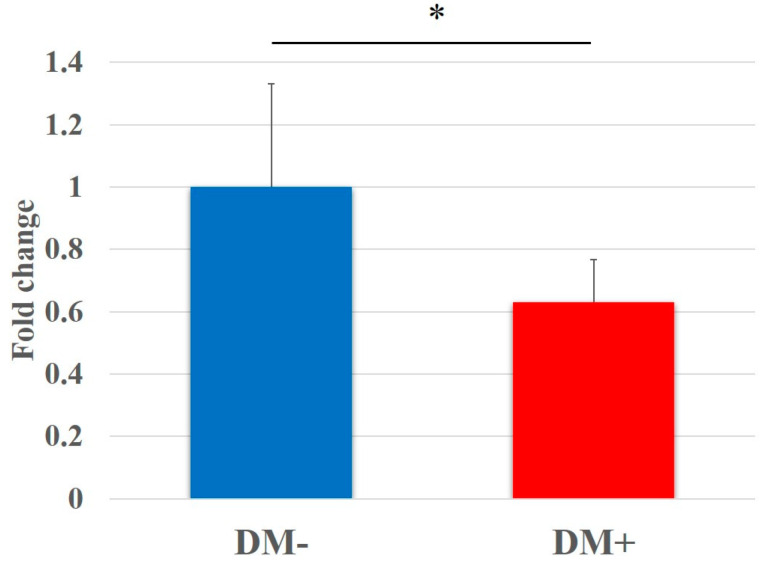
Proliferation of rotator-cuff-derived cells assessed using a water-soluble tetrazolium salt (WST)-based assay. Data are presented as the mean ± SD. The independent *t*-test was performed for determining significant differences: * *p* < 0.001. DM−, non-diabetic controls; DM+, diabetic group.

In the WST assay, the proliferation of rotator-cuff-derived cells cultured in the diabetic group for 48 h was shown to be significantly lower than that in the non-diabetic controls (DM−, 1.00 ± 0.31; DM+, 0.63 ± 0.14; *p* < 0.001). Fold changes in relative cell proliferation are shown in Figure 2.

### 3.3. qRT-PCR Analysis

**Figure 3:**
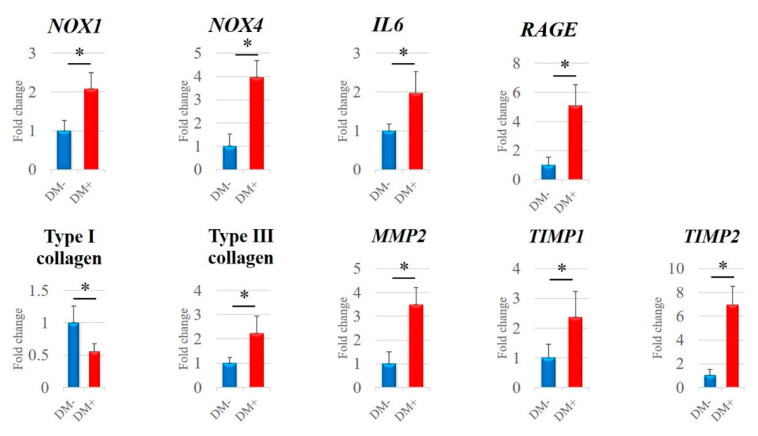
mRNA expression levels analyzed by quantitative reverse transcription polymerase chain reaction (qRT-PCR). *NOX1*, *NOX4*, *IL6*, *RAGE*, type I collagen, type III collagen, *MMP2*, *TIMP1*, and *TIMP2* mRNA expression levels were quantified in the diabetic group and non-diabetic controls. Data are presented as the mean ± SD. The independent t-test was performed for determining significant differences: * *p* < 0.05. NOX, nicotinamide adenine dinucleotide phosphate oxidase; IL, interleukin; RAGE, receptor for advanced glycation end-products; MMP, matrix metalloproteinase; TIMP, tissue inhibitor of matrix metalloproteinase.

The mRNA expression levels of *NOX1*, *NOX4*, *IL6*, *RAGE*, type III collagen, *MMP2*, *TIMP1*, and *TIMP2* in the diabetic group were significantly higher than those in the non-diabetic controls (*p* < 0.05; Figure 3). Contrarily, type I collagen expression was significantly lower in the diabetic group (*p* < 0.001; Figure 3).

### 3.4. Detection of ROS

**Figure 4:**
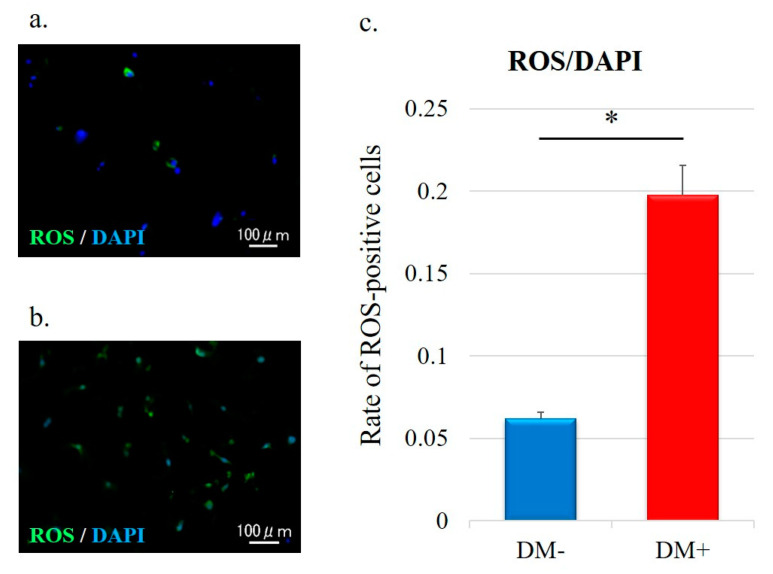
Representative immunofluorescence images of the detection of ROS levels in rotator-cuff-derived cells: (**a**) non-diabetic controls and (**b**) diabetic group. DCFH–DA staining (green) is indicative of the accumulation of reactive oxygen species (ROS) in rotator-cuff-derived cells; the nuclei are counterstained with 4′,6-diamidino-2-phenylindole (DAPI; blue). (**c**) Relative quantification of the ROS-positive cells (refers to Figure 4a,b). ROS-positive cells and DAPI-positive cells in four rectangular areas (0.75 mm × 1.0 mm) on each slide were counted and their mean values were analyzed. The ROS-positive cells rate (number of ROS-positive nuclei/DAPI-positive nuclei) is given as the mean of the four areas. Data are presented as the mean ± SD. The independent *t*-test was performed for determining significant differences: * *p* < 0.001. ROS, reactive oxygen species; DAPI, 4′,6-diamidino-2-phenylindole.

For determination of the actual oxidative status, intracellular ROS levels were measured by DCFH–DA staining; green staining was observed in the cytoplasm of ROS-positive cells (Figure 4a,b). The quantitative analysis of ROS-positive cells is shown in Figure 4c. The ROS-positive cell rate in the diabetic group was significantly higher than that in the non-diabetic controls (DM−, 0.06 ± 0.004; DM+, 0.20 ± 0.02; *p* < 0.001; Figure 4c).

### 3.5. Cell Apoptosis Analysis

**Figure 5:**
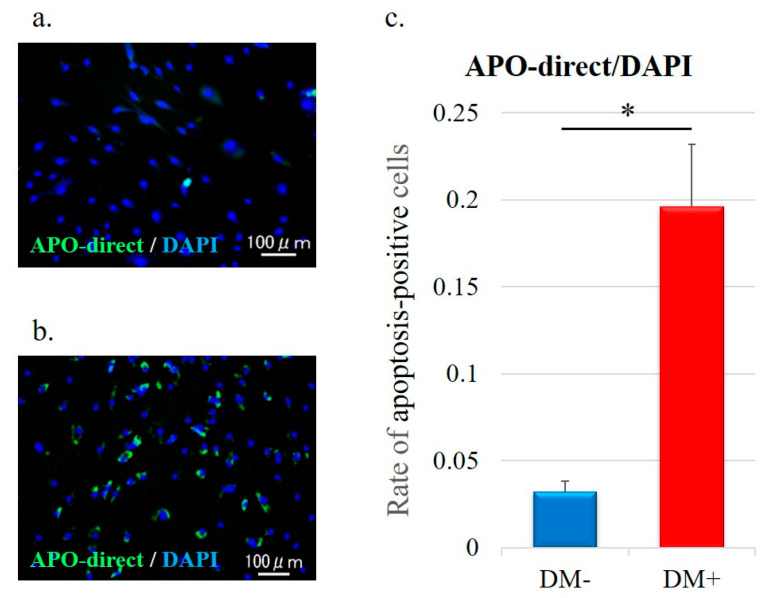
Representative immunofluorescence images of nuclear fragmentation (suggestive of apoptosis) in rotator-cuff-derived cells: (**a**) non-diabetic controls and (**b**) diabetic group. The TUNEL staining (green) highlights the apoptotic cells in each group. (**c**) Relative quantification of apoptotic cells. The number of apoptotic cells was analyzed by fluorescence intensity normalized to cell number. Apoptosis-positive cells and DAPI-positive cells in four rectangular areas (0.75 mm × 1.0 mm) on each slide were counted and their mean values were analyzed. The apoptosis-positive cells rate (number of apoptosis-positive nuclei/DAPI-positive nuclei) is given as the mean of the four areas. Data are presented as the mean ± SD. The independent *t*-test was performed for determining significant differences: * *p* < 0.001.

Detection of apoptotic cells was performed by TUNEL staining; fragments of the nuclei of apoptotic cells are stained green (Figure 5a,b). Quantitative analysis of apoptotic cells is shown in Figure 5c. The apoptotic cells rate in the diabetic group was significantly higher than that in the non-diabetic controls (DM−, 0.03 ± 0.006; DM+, 0.20 ± 0.04; *p* < 0.001; Figure 5c).

### 3.6. Rotator Cuff Histology and Immunohistochemistry

**Figure 6:**
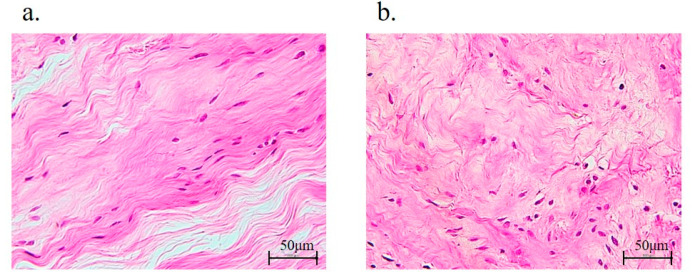
Rotator cuff histology. Hematoxylin and eosin staining of the supraspinatus tendons of non-diabetic controls (**a**) or diabetic group (**b**).

The rotator cuff (supraspinatus tendons) in the non-diabetic controls and diabetic group were evaluated histologically, and the latter showed significant abnormalities in fiber arrangement (DM−, 0.42 ± 0.50; DM+, 0.98 ± 0.79; *p* < 0.001; Table 3 and Figure 6). However, the fiber structure (DM−, 0.40 ± 0.49; DM+, 0.48 ± 0.50), nuclear morphology (DM−, 0.46 ± 0.50; DM+, 0.60 ± 0.57), and regional variations in cellularity (DM−, 0.28 ± 0.45; DM+, 0.36 ± 0.48) were not significantly different between the two groups (Table 3).

**Figure 7:**
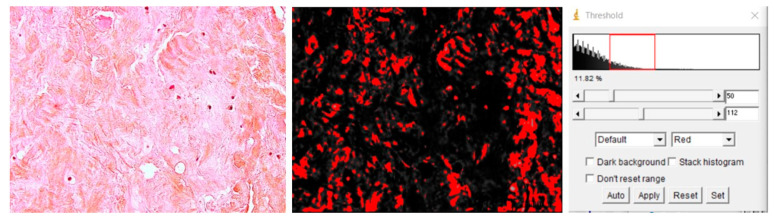
Quantitative evaluation of immunostaining using ImageJ. The percentage of regions stained red as positive regions for AGEs and RAGE staining was calculated.

**Figure 8:**
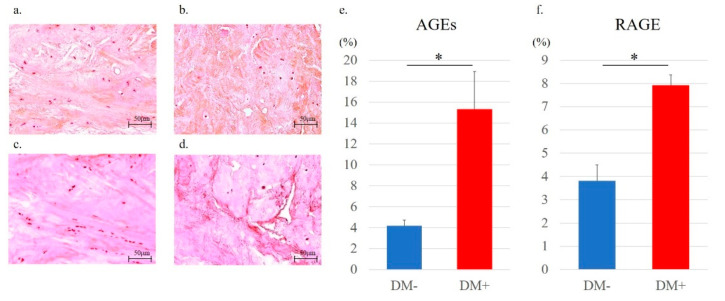
Immunohistochemistry staining for the detection of the expression of AGEs in the supraspinatus tendons in the non-diabetic controls (**a**) and diabetic group (**b**). The red-stained regions represent AGE-positive areas. Immunohistochemistry staining for the detection of the expression of RAGE in the supraspinatus tendons in the non-diabetic (**c**) and diabetic groups (**d**). The red-stained regions represent RAGE-positive areas. Quantitative analysis of the regions expressing AGEs (**e**) and RAGE (**f**). The percentages of AGEs and RAGE-positive regions were calculated by averaging the five selected fields at random per histological section. Data are presented as the mean ± SD. The independent *t*-test was performed for determining significant differences: * *p* < 0.001.

Quantitative evaluation of immunostaining was performed using ImageJ, according to a previous report (Figure 7) [26]. Immunostaining results showed that AGE-positive regions were mainly in the cytoplasmic matrix. Importantly, immunohistochemical staining of AGEs and RAGE revealed that the expression levels of AGEs and RAGE were significantly increased in the diabetic group (AGEs, DM− versus DM+; Figure 8a,b) (RAGE, DM− versus DM+; Figure 8c,d). Along with this result, the percentages of both AGEs (DM−, 4.18 ± 0.54; DM+, 15.31 ± 3.61; *p* < 0.001; Figure 8e) and RAGE (DM−, 3.82 ± 0.68; DM+ 7.92 ± 0.45; *p* < 0.001; Figure 8f) staining were significantly higher in the diabetic group than those in the non-diabetic controls.

## 4. Discussion

The present study investigated rotator cuffs in patients with non-traumatic shoulder rotator cuff tears and revealed that hyperglycemia caused by DM increased the mRNA expression level of *RAGE* and the protein levels of AGEs and RAGE. Moreover, ROS, apoptosis, and the mRNA expression of *NOX* was enhanced, and cell proliferation was decreased in rotator-cuff-derived cells in patients with DM. Altogether, these results highlight the influence of glycation and oxidative stress induced by DM in the human rotator cuff. To our knowledge, the present study is the first to report such effects.

DM is related to the increasing prevalence of common shoulder disorders, including shoulder pain, rotator cuff tendonitis, and rotator cuff tendon thickening [13]. Lin et al. also demonstrated that rotator cuff disorders, involving rotator cuff thickening and calcific tendinitis, occurred two-fold more frequently in patients with DM [27]. AGE accumulation in collagenous tissues of diabetic patients is considered to be one of the main factors of tissue dysfunction [11], which has been suggested as a mechanism for the development of diabetic complications such as nephropathy [28], retinopathy [29], and cataracts [30]. Mifune et al. have reported that AGEs also cause age-related degenerative rotator cuff changes by administering varying concentrations of AGEs to rotator-cuff-derived cells harvested from non-diabetic patients and observing the changes in the cells [12], but there have been no previous reports on the influence of diabetes-induced AGEs on the rotator cuff. In the present study, the expression levels of AGEs and RAGE were significantly increased in the rotator cuff of the diabetes patients, and the mechanisms related to oxidative stress were further investigated in detail as described below. Therefore, it may be useful to further elucidate the pathological conditions involving AGEs in diabetic patients. In addition, similar to previous reports of AGE loading on rotator-cuff-derived cells in vitro [12], the proliferation of rotator-cuff-derived cells was significantly reduced in the diabetic group, where AGE expression was higher.

Hyperglycemia caused by DM has also been shown to induce oxidative stress and cytokine production, leading to inflammation and tissue damage in various organs [31,32]. Oxidative stress is defined as the excessive production of toxic ROS in the body [33,34]. ROS are produced by various enzymes, including NOX, and mitochondrial electron transport systems under the control of growth factors and cytokines [35]. AGEs binding to RAGE also activate NOX, causing oxidative stress [10]. It is important to note that NOX-derived ROS are significant mediators of signaling pathways that regulate critical physiological responses such as cell growth, proliferation, migration, differentiation, apoptosis, and immune and biochemical reactions [36]. It has been demonstrated that *Nox1*, *Nox4*, and *Il6* mRNA expression was elevated in hyperglycemic rat tenocytes [37]. Additionally, it has been shown that overproduction of ROS promotes apoptosis, suggesting that important crosstalk exists for oxidative stress and apoptosis [38]. For example, oxidative stress in hyperglycemia has been demonstrated to induce apoptosis mediated by proapoptotic proteins [39]. This implies that injured or degenerated tendons might be impaired to recover by hyperglycemia. ROS have been shown to induce apoptosis of rotator cuff tenofibroblasts [40], which is related to rotator cuff tendon rupture [41,42]. Crucially, AGEs also increased ROS expression and the apoptotic rate in rotator-cuff-derived cells [12]. In the present study, ROS production and the apoptotic rate were significantly increased, as were *NOX1*, *NOX4*, and *IL6* mRNA expression levels in human rotator-cuff-derived cells of the diabetic group. There was no difference in BMI between groups while changes in oxidative stress were observed.

Collagen fiber morphology and stiffness changes were identified in Zucker diabetic Sprague-Dawley rat tail tendons [43]. The collagen fibers of diabetic tendons had insufficient organizing with no change in collagen content in a different histological analysis [44]. Additionally, the arrangement of collagen fibers in the rotator cuff with DM was also significantly disorganized in the present study.

Type I collagen constitutes about 90% of all normal tendon collagen, while type III collagen is more prevalent during inflammation [45]. It has been shown that patellar tendon type I collagen expression is suppressed by hyperglycemia [46]. Ueda et al., similarly described a decrease in type I collagen expression and an increase in type III collagen expression in the Achilles tendon under high-glucose conditions [5]. Here, we show that the expression of type I collagen was significantly decreased and that of type III collagen was significantly increased in the human rotator cuff of the diabetic group.

The metabolic activity of normal tendons is regulated by the expression balance between MMPs and TIMPs [47]. MMPs cleave inflammation-damaged interstitial collagen for remodeling, while TIMPs suppress MMP overexpression [47]. Of note, the production of MMP2 in adventitial fibroblasts is increased under the presence of high glucose levels [48]. In addition, increased mRNA expression of MMP2, TIMP1, and TIMP2 has been demonstrated for diabetic rat tendons. [5]. In the present study, diabetes upregulated *MMP2*, *TIMP1*, and *TIMP2* mRNA expression in the human rotator cuff.

This study had several limitations. First, the results of the subanalysis, which showed no significant correlation between the severity or duration of diabetes and the size of rotator cuff tears or the degree of fatty degeneration, may have been influenced by the small number of patients included in the study. It has been reported that the more severe the diabetes, the worse the degeneration of the Achilles tendon [49]. In this study, increased glycation and oxidative stress were also observed in the rotator cuff tissue in the diabetic group with higher HbA1c levels. Further studies with more patients are needed to investigate the correlation between the severity of diabetes and that of rotator cuff tears. Second, since this study evaluated the condition of the rotator cuff harvested at the time of the surgery, there is no indication of the actual impact of this condition on the rotator cuff tear. However, it is clear that the presence or absence of diabetes causes changes in rotator-cuff-derived cells and tissue due to the effects of glycation and oxidative stress, which may have an influence on rotator cuff tears.

## 5. Conclusions

We show that hyperglycemia caused by DM increases AGE and RAGE expression, which in turn increases NOX expression, ROS production, and apoptosis in the human rotator cuff. To our knowledge, this study is the first to demonstrate the influence of glycation and oxidative stress on the rotator cuff of humans with DM. These results indicate that non-traumatic rotator cuff tears in patients with diabetes are affected by glycation and oxidative stress. Therefore, the next challenge will be to find a preventive treatment for non-traumatic rotator cuff tears by inhibiting these effects.

## Figures and Tables

**Table 1:** Primer sequences for qRT-PCR analysis.

Gene	Oligonucleotide Sequence
*NOX1*	Forward 5′ GGTTTTACCGCTCCCAGCAGAA 3′ Reverse 5′ CTTCCATGCTGAAGCCACGCTT 3′
*NOX4*	Forward 5′ GCCAGAGTATCACTACCTCCAC 3′ Reverse 5′ CTCGGAGGTAAGCCAAGAGTGT 3′
*IL6*	Forward 5′ AGACAGCCACTCACCTCTTCAG 3′ Reverse 5′ TTCTGCCAGTGCCTCTTTGCTG 3′
*RAGE*	Forward 5′ CACCTTCTCCTGTAGCTTCAGC 3′ Reverse 5′ AGGAGCTACTGCTCCACCTTCT 3′
Type I collagen	Forward 5′ AGGAATTCGGCTTCGACGTT 3′ Reverse 5′ GGTTCAGTTTGGGTTGCTTG 3′
Type III collagen	Forward 5′ GGGAACAACTTGATGGTGCT 3′ Reverse 5′ CCTCCTTCAACAGCTTCCTG 3′
*MMP2*	Forward 5′ GTGCTGAAGGACACACTAAAGAAGA 3′ Reverse 5′ TTGCCATCCTTCTCAAAGTTGTAGG 3′
*TIMP1*	Forward 5′ CCAAGATGTATAAAGGGTTCCAA 3′ Reverse 5′ TTTCCAGCAATGAGAAACTCCT 3′
*TIMP2*	Forward 5′ GAGCCTGAACCACAGGTACCA 3′ Reverse 5′ AGGAGATGTAGCACGGGATCA 3′
*GAPDH*	Forward 5′ GTCTCCTCTGACTTCAACAGCG 3′ Reverse 5′ ACCACCCTGTTGCTGTAGCCAA 3′

NOX, nicotinamide adenine dinucleotide phosphate oxidase; IL, interleukin; RAGE, receptor for advanced glycation end-products; MMP, matrix metalloproteinase; TIMP, tissue inhibitor of matrix metalloproteinase; GAPDH, glyceraldehyde 3-phosphate dehydrogenase.

**Table 2:** Patient background characteristics.

	DM−	DM+	*p*-Value
Mean age	62.2 ± 6.4	65.3 ± 5.5	0.26
Sex	Men: 4; Women: 6	Men: 4; Women: 6	1
Mean HbA1c level (%)	5.7 ± 0.26	6.9 ± 0.5	<0.001 *
Duration of diabetes (years)	-	5.5 ± 4.4	-
Current pharmacotherapy	-	insulin: 2; oral medications: 4; insulin and oral medications: 2; none: 2	-
Prevalence of hypertension (%)	40	30	0.66
BMI (kg/m^2^)	24.5 ± 3.7	25.8 ± 3.5	0.44
Rotator cuff tear size (Cofield classification)	Small: 6; Medium: 3; Large: 1	Small: 6; Medium: 3; Large: 1	1
Fatty degeneration (Goutallier classification)	Stage 1: 5; Stage 2: 4; Stage 3: 1	Stage 1: 5; Stage 2: 4; Stage 3: 1	1

Mean values are presented as the mean ± SD. The independent t-test and chi-square test were used to determine significant differences (* *p* < 0.05). *n* = 10 in the non-diabetic controls (DM−) and diabetic group (DM+).

**Table 3:** Pathological score of tendons by hematoxylin and eosin staining.

	DM− Mean (SD)	DM+ Mean (SD)	*p*-Value
Fiber structure	0.40 (0.49)	0.48 (0.50)	0.43
Fiber arrangement	0.42 (0.50)	0.98 (0.79)	<0.001 *
Nuclear morphology (rounding)	0.46 (0.50)	0.60 (0.57)	0.20
Regional variations in cellularity	0.28 (0.45)	0.36 (0.48)	0.40

A score of 0 to 3 was used to evaluate each variable, with 0 = normal, 1 = slightly ab-normal, 2 = abnormal, and 3 = significantly abnormal. Rotator cuff tissues with H&E staining were graded ac-cording to five randomly selected optical fields for each histological section. Data are presented as the mean ± SD. An independent t-test was used to determine significant differences: * *p* < 0.05. *n* = 10 in the non-diabetic controls (DM−) and *n* = 10 in the diabetic group (DM+).

## Data Availability

All of the data is contained within the article.
